# COVID-19 Surface Persistence: A Recent Data Summary and Its Importance for Medical and Dental Settings

**DOI:** 10.3390/ijerph17093132

**Published:** 2020-04-30

**Authors:** Luca Fiorillo, Gabriele Cervino, Marco Matarese, Cesare D’Amico, Giovanni Surace, Valeria Paduano, Maria Teresa Fiorillo, Antonio Moschella, Alessia La Bruna, Giovanni Luca Romano, Riccardo Laudicella, Sergio Baldari, Marco Cicciù

**Affiliations:** 1 Department of Biomedical and Dental Sciences, Morphological and Functional Images, University of Messina, Azienda Ospedaliera Universitaria “G. Martino”, Via Consolare Valeria, 98100 Messina, Italy; gcervino@unime.it (G.C.); matamarco94@gmail.com (M.M.); cdamico@unime.it (C.D.); sbaldari@unime.it (S.B.); 2 Clinical Analysis Laboratory “Dott. Francesco Siracusa Rizzi s.r.l.”, Via Nazionale Archi, 89121 Reggio Calabria, RC, Italy; giox84@hotmail.com; 3 Unit of Microbiology and Virology, North Health Center ASP 5, 89100 Reggio Calabria, RC, Italy; valeria.paduano25@virgilio.it (V.P.); mariatfiorillo@hotmail.com (M.T.F.); 4 Azienda Ospedaliera Bianchi-Melacrino-Morelli, 89100 Reggio Calabria, RC, Italy; moschellan@gmail.com; 5 IRCCS San Raffaele Scientific Institute, Milan 20132, Italy; Labruna.alessia@hsr.it; 6 Department of Biomedical and Biotechnological Sciences, School of Medicine, University of Catania, 95100 Catania, Italy; giovanniluca.romano@unict.it; 7 Nuclear Medicine Unit, Department of Biomedical and Dental Sciences and Morpho-Functional Imaging, University of Messina, 98100 Messina, Italy; riclaudi@hotmail.it

**Keywords:** COVID-19, virus, epidemiology, surfaces, infection risk

## Abstract

Recently, due to the coronavirus pandemic, many guidelines and anti-contagion strategies continue to report unclear information about the persistence of coronavirus disease 2019 (COVID-19) in the environment. This certainly generates insecurity and fear in people, with an important psychological component that is not to be underestimated at this stage of the pandemic. The purpose of this article is to highlight all the sources currently present in the literature concerning the persistence of the different coronaviruses in the environment as well as in medical and dental settings. As this was a current study, there are still not many sources in the literature, and scientific strategies are moving towards therapy and diagnosis, rather than knowing the characteristics of the virus. Such an article could be an aid to summarize virus features and formulate new guidelines and anti-spread strategies.

## 1. Introduction

### 1.1. Rationale

Coronavirus disease 2019 (COVID-19) is an infectious respiratory disease caused by the virus called severe acute respiratory syndrome coronavirus 2 (SARS-CoV-2), belonging to the coronavirus family. An infected person may experience symptoms after an incubation period that could vary from about 2 to 14 days (there have rarely been cases of incubation periods of 29 days), during which time the person could still be contagious. To limit transmission, precautions should be taken, such as adopting careful personal hygiene, washing hands frequently and wearing masks. Coronavirus mainly affects the lower respiratory tract and causes a number of symptoms described as flu-like, including fever, cough, shortness of breath, muscle pain, tiredness and gastrointestinal complaints such as diarrhea. In severe cases, pneumonia, acute respiratory distress syndrome, sepsis and septic shock could occur, up to the death of the patient. Among the collective preventive measures, it should be noted that in 2003, during the SARS epidemic, the major collective catering companies in China and Hong Kong adopted the obligation to wear surgical masks for their service personnel to protect both the workers of companies and the public. This professional category is particularly exposed to potentially infectious contacts, both active and passive. In the same way, a person in charge of equipment who is not equipped with the appropriate personal protective equipment (PPE) will find themselves exposed to contact with the dirty dishes and recent food remains of a large number of customers. Further interventions in the restaurant sector may include a prohibition on the distribution of buffets for both food and dishes [1,2,3,4].

In recent weeks there has been about a great deal of discussion about the contamination and decontamination of inanimate surfaces. In fact, the duration before inactivation of the COVID-19 virus on surfaces (liquid, solid or gaseous) is still debated. In most cases, the spread between people occurs through the respiratory droplets emitted by an infected individual through coughing or sneezing, which, are subsequently inhaled by a healthy person who is nearby. This also caused an initial diffidence on the part of people in purchasing products of any kind coming (including by post) from the areas affected by the epidemic, leading to economic damage. It is possible to become infected by touching surfaces or objects where the virus is present and then bringing your hands towards your mouth, nose or eyes [5,6,7,8,9,10]. In ideal conditions, the virus can in fact persist on different surfaces for hours or days. The surfaces most exposed to this type of transmission include, for example:

- Door handles;
- Lift or light buttons;
- Mobile phones;
- Public transport handholds [11,12,13,14].

The viral titration, viral assay or viral count is the count, made in the laboratory, of the number of viral particles of a given virus under examination present in a biological sample. It is used in microbiological research, diagnostics and in the production of antiviral vaccines—all situations that require knowledge of the amount of virus being analyzed or used. We used tissue-culture infectious dose (TCID) per milliliter for COVID-19 persistence evaluation [15,16,17,18,19].

### 1.2. Objectives

The aim of this article is to evaluate, through the analysis of the current literature, how long this virus can remain active on different surfaces. It is too early to be able to carry out a review with meta-analysis of the literature given the incredible relevance of this topic, but it is certainly a step to clarify this pandemic.

The following questions were used to develop the study framework according to the PICO (Population/Intervention/Comparison/Outcomes) guidelines:

- What is the persistence of SARS-CoV-2 on surfaces?
- What is the mean persistence of coronaviruses compared to SARS-CoV-2?

## 2. Methods

### 2.1. Protocol and Registration

An investigation methods protocol was used, according to the PRISMA statement; the aim of the PRISMA statement is to help authors improve the reporting of systematic reviews and meta-analyses. It can be used as a basis for reporting reviews of other types of research. The use of checklists like PRISMA is likely to improve the reporting quality of a systematic review and provides substantial transparency in the selection process of papers in a systematic review. Furthermore, PICO guidelines have been used to prepare summary questions [20,21,22].

### 2.2. Eligibility Criteria

The full texts of all studies related to the main revision topics were obtained for comparing the inclusion parameters:

- SARS-CoV-2 features articles;
- SARS-CoV-2 persistence;
- The persistence of other coronaviruses.

The following were the exclusion criteria:

- Not enough information regarding the topic;
- Articles published prior to 1 January 2010;
- No access to the title and abstract.

### 2.3. Information Sources

Research was conducted in five electronic databases, including MEDLINE, PubMed, and EMBASE. In addition, a manual search was conducted for relevant studies published.

### 2.4. Search

Digital and manual searches were then performed. The data search was performed in order to add significant studies and to increase the sensitivity of this study. For the search we used keywords according to Medical Subject Headings (MeSH). (SARS-CoV-2 OR Coronavirus) AND (Persistence OR surface) terms were investigated on information sources, as specified in Section 2.3.

### 2.5. Study Selection

First, the manuscript titles list was highlighted to exclude irrelevant publications and search errors. The final selection was performed by reading the full texts of the papers in order to approve each study’s eligibility based on the inclusion and exclusion criteria.

### 2.6. Data Collection Process

Data selection and revision was performed by independent reviewers of three different affiliations (Luca Fiorillo, Messina; Giovanni Surace, Reggio Calabria; Alessia La Bruna, Milan). They singularly analyzed the obtained papers. Results obtained were compared and discussed with a fourth independent reviewer (Gabriele Cervino, University of Messina) when a consensus could not be reached. For the stage of the full-text articles’ revision, a complete independent analysis was performed.

### 2.7. Data Items

Results were singularly analyzed and items about viruses’ persistence on different materials were evaluated and shown.

We created a table to show a summary of inherent virus features.

Table 1 items are as follows:

- Virus: type of investigated virus;
- Authors and Year: authors, reference (according to journal guidelines) and year of publication;
- Investigated Material: investigated material surfaced by the study;
- Time: persistence time of virus;
- Note on Results: additional notes on results.

### 2.8. Risk of Bias

The grade of bias risk was independently considered, as reported in [23,24,25].

Potential causes of bias were investigated:

- Selection bias;
- Performance bias and detection bias;
- Attrition bias;
- Reporting bias;
- Examiner blinding, examiner calibration, standardized follow-up description, standardized residual graft measurement, standardized radiographic assessment.

### 2.9. Synthesis of Results

We conducted a manual synthesis of article results.

## 3. Results

### 3.1. Study Selection

**Figure 1:**
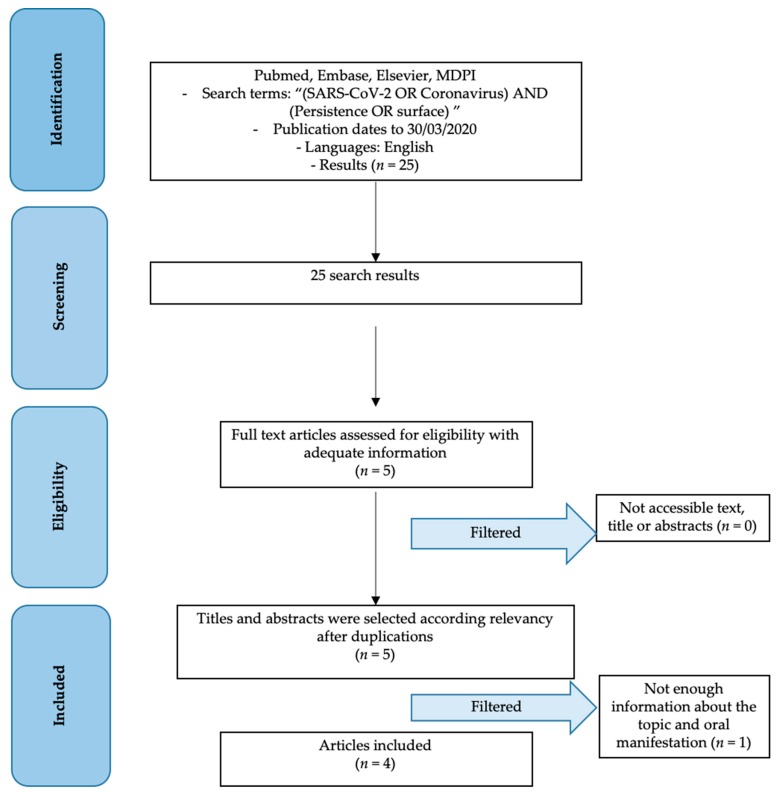
PRISMA checklist.

During the first search, 25 studies were obtained. After applying inclusion and exclusion criteria, only the remaining 5 articles were further analyzed. Then, articles were manually selected, and finally 4 articles were obtained (Figure 1). 

### 3.2. Results of Individual Studies

Results of individual studies were shown after an accurate analysis in Table 1. The aim of this table is to summarize coronaviruses’ persistence time.

**Table 1:** Results of individual studies. Results are organized as described in Section 2.7.

Virus	Authors and Year	Investigated Material	Time	Note on results
2019-nCoV	Van Doremalen et al. 2020 [26]	aerosols	3 h	Reduction from 10^3.5^ to 10^2.7^ TCID_50_ per liter of air
		plastic	72 h	Reduction from 10^3.7^ to 10^0.6^ TCID^50^ per millimeter
		stainless steel	48 h	from 10^3.7^ to 10^0.6^ TCID^50^ per millimeter
		copper	4 h	No viable SARS-CoV-2
		cardboard	24 h	No viable SARS-CoV-2
Other coronaviruses	Van Doremalen et al. 2020 [26]	aerosols	3 h	reduction from 10^4.3^ to 10^3.5^ TCID_50_ per liter of air
		plastic	72 h	from 10^3.4^ to 10^0.7^ TCID^50^ per millimeter
		stainless steel	48 h	from 10^3.6^ to 10^0.6^ TCID^50^ per millimeter
		copper	8 h	No viable SARS-CoV-1
		cardboard	8 h	No viable SARS-CoV-1
	Kampf et al. 2020 [27]	paper	5 min up to 5 days	10^5^ TCID^50^ per millimeter
		glass	4–5 d	10^4^ TCID^50^ per millimeter
		plastic	2–9 d	10^6^ TCID^50^ per millimeter
		PVC	5 d	10^3^ TCID^50^ per millimeter
		silicon rubber	5 d	10^3^ TCID^50^ per millimeter
		surgical gloves (latex)	5 d	10^3^ TCID^50^ per millimeter
		disposable gowns	1–2 d	10^5^ TCID^50^ per millimeter
	Warnes et al. 2015 [28]	polyfluorotetraethylene (PTFE)	5 d	10^3^ TCID^50^ per millimeter
		ceramic	5 d	10^3^ TCID^50^ per millimeter
		glass	5 d	10^3^ TCID^50^ per millimeter
		stainless steel	5 d	10^3^ TCID^50^ per millimeter
		polyvinyl chloride (PVC)	5 d	10^3^ TCID^50^ per millimeter
		silicon rubber	3 d	10^3^ TCID^50^ per millimeter
		brasses containing copper	<40 min	10^3^ TCID^50^ per millimeter
		copper nickels	120 min	10^3^ TCID^50^ per millimeter
		zinc	60 min	

### 3.3. Synthesis of Results

Van Doremalen et al. [26] evaluated the stability of SARS-CoV-2 and SARS-CoV-1 in aerosols and different surfaces. They evaluated these viruses’ decay rates using Bayesian linear regression. They conducted their experiment using aerosols (< 5 μm) containing SARS-CoV-2 (105.25 50% tissue-culture infectious dose (TCID50) per milliliter) or SARS-CoV-1 (106.75-7.00 TCID50 per milliliter) generated by a nebulizer. Ten different experimental conditions involving SARS-CoV-1 or 2 were evaluated. Results on infectious titer reduction are shown in Table 1. The data were expressed as 50% tissue-culture infectious dose (TCID_50_). It is important to specify that the limit of detection for this experiment was 3.33 × 10^0.5^ TCID_50_ per liter of air for aerosols; 10^0.5^ TCID_50_ per milliliter of medium for plastic, steel, and cardboard; and 10^1.5^ TCID_50_ per milliliter of medium for copper. Kampf et al. [27] showed how different coronaviruses could persist on different types of inanimate surfaces. They also evaluated some environmental characteristics, such as temperature or humidity. They showed how human coronavirus could be influenced by temperature, as 30 or 40 °C reduced the duration of persistence of coronaviruses on inanimate surfaces. However, at the temperature of 4 °C, the persistence could be greater than or equal to 28 days. Another important result is that the persistence was longer with higher inocula. Warnes et al. [28] evaluated coronaviruses’ persistence on metal and non-metal samples. They inoculated 10^3^ plaque forming units (PFU) on different materials: polyfluorotetraethylene (Teflon; PTFE), polyvinyl chloride (PVC), ceramic tiles, glass and stainless steel. Coronaviruses’ persistence was at least 5 days (and 3 days for silicon rubber) at 21 °C. Despite this, it could be rapidly inactivated by brass and copper nickel surfaces in less than 60 min. Copper nickel surfaces were effective but less than brass copper; in these cases, the inactivation time was up to 5 min in the fingertip contamination model. Warnes et al. demonstrated how a higher percentage of copper could lead to superior antiviral properties. Another important factor reported in this study was that the release of ions from copper and the formation of reactive oxygen species (ROS) take part in the deactivation of the virus. Furthermore, the authors report that following an analysis carried out with a transmission electron microscope (TEM), the virus was normally present on common surfaces such as stainless steel, but on copper surfaces it appeared to be damaged and intact particles were few.

### 3.4. Risk of Bias

It was not possible to conduct a bias risk analysis according to the PRISMA statement as specified in the previous section. Unfortunately, the limited number of articles obtained does not allow for the realization of a systematic review. The individual studies were analyzed, and the experiments leading to the results shown in Table 1 were rigorously conducted and are repeatable analyses [29].

## 4. Discussion

### 4.1. Summary of Evidence

The literature concerning the characteristics of these viruses is still scarce, especially if one considers only COVID-19. The aspects related to the persistence of the virus on surfaces not only represent an environmental and public health problem concerning schools, roads, offices. It is a much bigger problem if hospitals, operating theaters, and sanitary waiting rooms are considered, especially in the new and continuously increasing “COVID departments”. Knowing how the virus behaves in contact with surfaces and with different disinfectants could be important for the sanitization of medical environments. In particular, some authors deal with the optimization of infection control in operating rooms. Some devices are used in hospital operating rooms for single use only, but other devices, surfaces, handles and cords could be transmission vehicles [30]. Ong et al. 2020 [31] evaluated the presence of coronavirus in a hospital room of COVID-19 patients. Some surfaces, such as the toilet bowl and the sink, were positive. Room air samples and samples collected after cleaning were negative. The time span varied according to the characteristics of the type of surface: the less-porous ones like plastic and steel were the worst because they absorb droplets less easily, preserving the active virus. Additionally, the different environmental conditions could affect the amount of ventilation of the rooms and the humidity [26]. According to Van Doremalen et al. [26], aerosol and surface virus transmission is plausible, since it can remain viable and infectious for hours or days. Kampf et al. [27] showed how human coronaviruses can remain infectious on surfaces for up to 9 days at room temperature. This is an important factor about coronaviruses’ spread. From this, it is easy to see that if someone tends to touch the environment often—especially if not properly disinfected—the possibility of becoming infected increases. Furthermore, the droplets present in the form of an aerosol of an infected patient can not only easily spread, but also easily settle and last for several hours on a surface. Kampf et al. [27] investigated different biocidal agents on coronaviruses. They demonstrated how ethanol (78%–95%), 2-propanol (70%–100%), the combination of 45% 2-propanol with 30% 1-propanol, glutardialdehyde (0.5%–2.5%), formaldehyde (0.7%–1%) and povidone iodine (0.23%–7.5%) readily inactivated coronavirus infectivity by approximately 4 log_10_ or more. Sodium hypochlorite required a minimal concentration of at least 0.21% to be effective. Hydrogen peroxide was effective with a concentration of 0.5% and an incubation time of 1 min. An important finding is the ineffectiveness of chlorhexidine. Within 10 min, a concentration of 0.2% revealed no efficacy against coronavirus. It is a result that does not support some guidelines for dentistry [32]. Kampf et al. [27] concluded that these viruses can remain on surfaces up to 9 days and that surface disinfection could be performed with 0.1% sodium hypochlorite or 62%–71% ethanol for 1 minute. According to Warnes et al. [26], coronavirus persists in an infectious state on surfaces for several days. Warnes et al. [26] demonstrated the survival of coronaviruses on different surfaces for up to 5 days. Concerning the persistence of the virus on different surfaces, and in particular on metals containing copper, these findings are interesting and could lead to the development of new surfaces with viricidal or bactericidal properties. In confined environments, especially if poorly ventilated, viral particles of less than 0.1 µm in size may remain in the environment as a secondary aerosol. Studies on the topic indicate that a sneeze could release up to 2 million droplets into the air, less than a million from a cough and about 3000 from speaking out loud. The droplets eliminated from the airways, if larger than 100 µm, from a height of 2 m settle on flat surfaces in 3–6 s and reach horizontally about 1.5 m away, then evaporate rapidly, dry and become solid material. This material reaches a size of 2–3 µm. Studies on tuberculosis have shown that this material, maintaining its infectious capacity, could be inhaled and, thanks to its size, reach the most peripheral parts of the lungs, becoming a secondary biological aerosol [26]. There has not been much discussion about the importance of ventilating environments to prevent SARS-CoV-2 infection, and although the viral particles have not been studied sufficiently for their ability to achieve dangerous concentrations from a distance in confined environments, increased ventilation in an environment is believed to reduce the cross-infection of airborne diseases. Therefore, existing recommendations could be amended to include ventilating public spaces, including means of transport, with suitable means. It is essential to focus on preventing infection by using ventilation suitable to reduce the infectious capacity of the coronavirus. It has been widely demonstrated that natural ventilation causes better air exchange compared to mechanical ventilation—up to 69 changes per hour of rooms when the windows are completely open [33]. Most international guidelines recommend about 12 changes per hour for isolation rooms in case of infections. Medical and dental staff will have to work safely, and this is complicated by the fact that there are still no official guidelines that tell them how to behave.

Moreover, as suggested by Spagnuolo et al., dentists should avoid the scheduling of any patient: only such urgent dental diseases can be considered during the COVID-19 outbreak. The waiting time of patients in dental offices could highly predispose patients to be infected. When dentists treat patients, they should intercept potentially infected persons before they reach the operating areas; for example, identifying those with a fever measuring >37.5 °C and posing a few questions about the patient’s general health status in the last 7 days, and about the risk of having been in contact with other infected persons. Dentists as well as medical office employees should always be covered by facial masks, and they should remind patients to maintain the 1-m distance with each other during their waiting time [34,35].

Disinfecting surfaces is one of the aspects to which to give greater attention, being done with the detergents already in use today, along with the washing of hands and the use of suitable PPE. In regards to PPE, surgical masks must be used by those who could transmit the virus, but those who work in contact with the patient’s aerosol must use FFP2 and FFP3 (Filtering Facepiece Particles) masks. There should only be one patient in waiting rooms. This is also true in clinical areas; in the case of minors who need an escort, the escort must be at a distance from operators, and must wear a surgical mask. At the end of each session, the surfaces will then have to be cleaned, and the air exchanged. The same procedures should be adopted in the waiting room and in other areas where the patient might pass or touch objects. Certainly, some tools, such as quick tests (once validated), can become useful in the hands of the doctor/dentist to understand if the patient, or some member of their team, is potentially infected.

### 4.2. Limitations

We are aware of the limitations of this article; it is not possible to make a review of the literature due to the lack of data. So, this is only a summary of the available information. Further studies are needed in order to verify the persistence times of coronavirus on surfaces more precisely. However, we have considered proceeding with the creation of this summary, while respecting the guidelines of systematic reviews and also not having sufficient numbers. The reason for this choice lies in the incredible topicality and reliability of the data obtained, with the hope that this work could be widely and immediately used to contain the pandemic.

## 5. Conclusions

The purpose of this summary is to draw a line and clarify the characteristics of SARS-CoV-2 with regard to its persistence on surfaces. This article is of great interest and could be used to write new guidelines by epidemiologists, having a clear summary of the current situation. From our analysis it is possible to deduce some aspects. The virus can reach surfaces in the form of an aerosol. Therefore, following nebulization through people (sneezing or coughing) or electromedical machinery, infection via surfaces should be considered, since the latter could remain viable and infectious for hours or days. On average, the different coronaviruses persist in an infectious state on surfaces for several days, even up to nine. Surface disinfection could be performed with 0.1% sodium hypochlorite or 62%–71% ethanol for 1 minute. Copper has shown antiviral properties—so much so that the virus appears damaged or altered on copper surfaces.

Other experiments are certainly needed, on different surfaces or even on biological surfaces, to better understand the persistence times of this virus and promote adequate standards.
